# Representing core gene expression activity relationships using the latent structure implicit in Bayesian networks

**DOI:** 10.1093/bioinformatics/btae463

**Published:** 2024-07-25

**Authors:** Jiahao Gao, Mark Gerstein

**Affiliations:** Program in Computational Biology and Bioinformatics, Yale University, New Haven, CT 06520, United States; Program in Computational Biology and Bioinformatics, Yale University, New Haven, CT 06520, United States; Department of Molecular Biophysics and Biochemistry, Yale University, New Haven, CT 06520, United States; Department of Statistics and Data Science, Yale University, New Haven, CT 06520, United States; Department of Computer Science, Yale University, New Haven, CT 06520, United States

## Abstract

**Motivation:**

Many types of networks, such as co-expression or ChIP-seq-based gene-regulatory networks, provide useful information for biomedical studies. However, they are often too full of connections and difficult to interpret, forming “indecipherable hairballs.”

**Results:**

To address this issue, we propose that a Bayesian network can summarize the core relationships between gene expression activities. This network, which we call the LatentDAG, is substantially simpler than conventional co-expression network and ChIP-seq networks (by two orders of magnitude). It provides clearer clusters, without extraneous cross-cluster connections, and clear separators between modules. Moreover, one can find a number of clear examples showing how it bridges the connection between steps in the transcriptional regulatory network and other networks (e.g. RNA-binding protein). In conjunction with a graph neural network, the LatentDAG works better than other biological networks in a variety of tasks, including prediction of gene conservation and clustering genes.

**Availability and implementation:**

Code is available at https://github.com/gersteinlab/LatentDAG

## 1 Introduction

Understanding gene regulation is important for biological research. The highly interconnected relationships between genes form various types of biological networks. For example, chromatin immunoprecipitation followed by sequencing (ChIP-seq) is efficient in identifying proteins that bind to different genome regions. This allows us to establish a connection between transcription factors (TFs) and their target genes, eventually forming the gene regulatory network (GRN). Physical interactions between different proteins can be characterized as protein–protein interactions (PPI), and correlations in gene expression are represented as a co-expression network. One gene could bind to the DNA of another gene while physically interacting with many others. These intricate regulations together make up the complex biological system.

To date, researchers have generated a large amount of different biological networks. For example, BioGRID v4.4.220 (Stark *et al.* 2006) provides a collection of 2.6M interactions identified from 82 024 publications. However, to fully utilize these biological networks, their quality is essential. False and missing network connections likely impair most analyses on the networks. Furthermore, biological networks such as ChIP-based GRN usually represent homogeneous relationships, limiting our capability to fully understand or interpret the results. Finally, many complex networks do not match the conditions in which they are employed, and this discrepancy can be problematic. It is known that gene regulation is a dynamic process, and a large amount of network rewiring happens when the same cells are in different circumstances (Li and Johnson 2010).

Graph-based algorithms have been used to leverage biological networks in biomedical studies. For example, researchers used network propagation to identify key genes relevant to cancer (Mohsen *et al.* 2021). However, the intrinsic complexity of the networks often impedes further analysis. For example, simulated annealing could be used as a compromise to find the global optimum when optimizing the graph layout (Cheng *et al.* 2015). However, this type of algorithm suffers from its poor scalability and may fail to reach the optimum in practice (Guilmeau *et al.* 2021). Among graph-based models, graph neural networks (GNNs) have recently gained widespread popularity. Particularly, the graph convolutional networks (GCNs) (Kipf and Welling 2017) have shown a great capability in effectively integrating the graph structure together with the node features to make accurate predictions in various fields. For each node, a GCN layer essentially aggregates the information from its neighbors and converts its original node feature vector to an embedding vector, usually of a different number of dimensions. By connecting *N* such layers, the model effectively incorporates information from all nodes within *N* steps away. The final classification or prediction is then achieved by feeding the final embedding to a fully connected layer.

To alleviate the problems in biological networks, we proposed that a network structure that learned directly from the gene-level data might be a more accurate representation of the underlying relationships between genes. Because the GNN models efficiently leverage graph structure and provide easily evaluable measurements, we sought to demonstrate that the learned network can improve the performance of GNN models in various tasks. Practically, we relied on the Bayesian network structure learning methods. A Bayesian network is a well-studied model to describe the joint probability distribution for a set of variables (Scanagatta *et al.* 2019) and is often used for causal inference. A Bayesian network has a directed acyclic graph (DAG) structure, and every variable in the network is conditionally independent of its nondescendants given its parents. After assigning local probability distributions in the network, interesting questions, such as the probability of having a certain disease given the symptoms, could be answered. In fact, any marginal or conditional probabilities can be computed, and this process was also considered as causal inference. However, Bayesian network structure learning is computationally expensive, as the number of combinations grows super-exponentially with the number of nodes (Scanagatta *et al.* 2019). There have been two major types of approaches to solving the Bayesian network structure learning problem. In a score-based approach, researchers first define a score function to evaluate how well a Bayesian network fits the data, then search over the DAG space to find the best network. In a constraint-based approach, independence relationships between nodes were first tested to identify a set of edge constraints. Researchers then performed a similar search to find the best DAG satisfying the constraints. Interestingly, a recently developed method, NOTEARS (Zheng *et al.* 2018), provides a different solution. NOTEARS proposed a simple continuous function as the constraint, thus converting the whole problem from a combinatorial searching problem into a numerical optimization problem. This allows us to make use of existing optimization techniques and enables us to work on large datasets. Following the success of NOTEARS, many similar methods have been developed, including DAG-GNN (Yu *et al.* 2019), No Fears (Wei *et al.* 2020), DAGMA (Bello *et al.* 2022), etc., with each having a slightly different constraint function. For example, DAGMA used a different continuous constraint function to better detect large cycles, have better-behaved gradients, and reduce the runtime significantly (Bello *et al.* 2022). Regardless of the difference, all these tools provide a Bayesian network which serves as a simplified representation of the underlying relationships. We focus on one of the newest tools, DAGMA, as well as the earliest one, NOTEARS, for our analysis.

In this article, we first applied the Bayesian network structure learning methods on a gene expression dataset. We found that the resulting network, which we called the LatentDAG, provided a much more simplified and interpretable version of gene activity relationships by presenting the backbone of the tangly co-expression “hairballs.” When combined with GNN models, we found that Bayesian networks outperformed other existing biological networks such as ChIP-based GRN, PPI, or co-expression in a variety of tasks. We detailed a few representative examples such as prediction of gene conservation levels and gene clustering using the GNN embeddings. Further analysis of the Bayesian network revealed that, despite being relatively simple, this core network captured biologically significant interactions of various types.

## 2 Materials and methods

### 2.1 Expression data

We collected the expression data from a published perturb-seq dataset (Replogle *et al.* 2022). Specifically, the researchers knocked down a single gene by CRISPR and then measured the expression of all genes by a follow-up single-cell RNA-seq. In the K562 cell line, the authors identified 1973 perturbations with strong phenotypes and 2319 highly variable genes. We collected the Z-normalized gene expression value for these 2319 genes across 1973 perturbations directly from the paper. This dataset not only provides a series of uniformly processed data under a large number of conditions but also provides a wider range of the expression of each single gene, making it easier to capture possible regulations.

### 2.2 Network data

We used DAGMA (https://github.com/kevinsbello/dagma) (Bello *et al.* 2022) to infer the network structure between the genes. Specifically, DAGMA estimates a continuous adjacency matrix *W* by solving the following problem:

(1) $$\min_{W\in R^{d\times d}}\frac{1}{2n}{\left\|X-XW\right\|}^{2}+\lambda {\left\|W\right\|}_{1}$$

(2) subject to h(W)=−log det(sl−W⊙W)+d log s=0

where *X* is the samples by genes matrix, *d* is the number of genes, *λ* is the L1-regularization parameter, det is the matrix determinant, *I* is the identity matrix of size *d*, s is a given real number (e.g. 1 by the program default), and ⊙ is the element-wise Hadamard product. Then, the binary adjacency matrix *A* is defined by [*A*(*W*)]_*ij* = 1 if and only if *w*_*ij* ≠ 0. To solve the above numerical optimization problem, DAGMA adapts a path-following approach, where the key idea is to use the previous solution as the starting point for the current unconstrained problem. Finally, DAGMA set a fixed threshold on *W* to remove noise and small numbers during optimization calculations. To identify more relationships, we used an adaptive threshold instead by slowly decreasing the threshold until the graph based on *W* was no longer a DAG. We called the graph *A* based on the final *W* a LatentDAG. Similarly, we ran the program NOTEARS (https://github.com/xunzheng/notears) (Zheng *et al.* 2018) with default parameters to infer a second DAG from the data.

For ChIP-seq-based GRNs, we collected the TF–target relationships from the hTFtarget database (Zhang *et al.* 2020). Alternatively, we downloaded the processed bed file of 500 ChIP-seq experiments from different human cell lines from the ENCODE portal (Jou *et al.* 2019). We then ran TIP (Cheng *et al.* 2011) and Storey and Tibshirani multiple testing correction (Storey and Tibshirani 2003) to identify significant TF–target relationships. Additional filtering on the ENCODE ChIP-seq dataset resulted in a K562 cell-line-specific subnetwork.

For the co-expression network, we first calculated the correlation of gene expression across 1973 perturbations for the 2319 genes. Alternatively, we downloaded the gene expression values of whole blood samples from GTEx (GTEx Consortium 2020) and calculated the expression correlation across the GTEx samples. Finally, we collected all available total RNA-seq data for the K562 cell line from the ENCODE portal. We applied a threshold of 0.5 and 0.75 on the absolute value of the correlation for the perturb-seq data and GTEx, respectively. These thresholds were chosen because they were the turning point on the threshold vs. number-of-edge curve (Supplementary Fig. S11). There was no clear turning point for the ENCODE expression, so we used the threshold 0.75 as well.

Finally, we downloaded the gene–gene relationships from the BioGRID database (BIOGRID-ORGANISM-Homo_sapiens-4.4.218.tab3.txt) (Stark *et al.* 2006, Oughtred *et al.* 2019) and STRING database (9606.protein.physical.links.v11.5.txt) (Szklarczyk *et al.* 2019). We limited the BioGRID dataset to physical interactions only by filtering “Experimental System Type” equal to “physical.”

All the above networks were filtered to include only the 2319 genes that existed in the perturb-seq dataset.

In addition, we simulated two random networks, using the Erdős–Rényi model and scale-free model, respectively, with the same size as the ChIP GRN from hTFtarget. This was carried out using the utility functions included in the DAGMA package.

### 2.3 Analysis of the LatentDAG genes and edges

**Figure 1.**
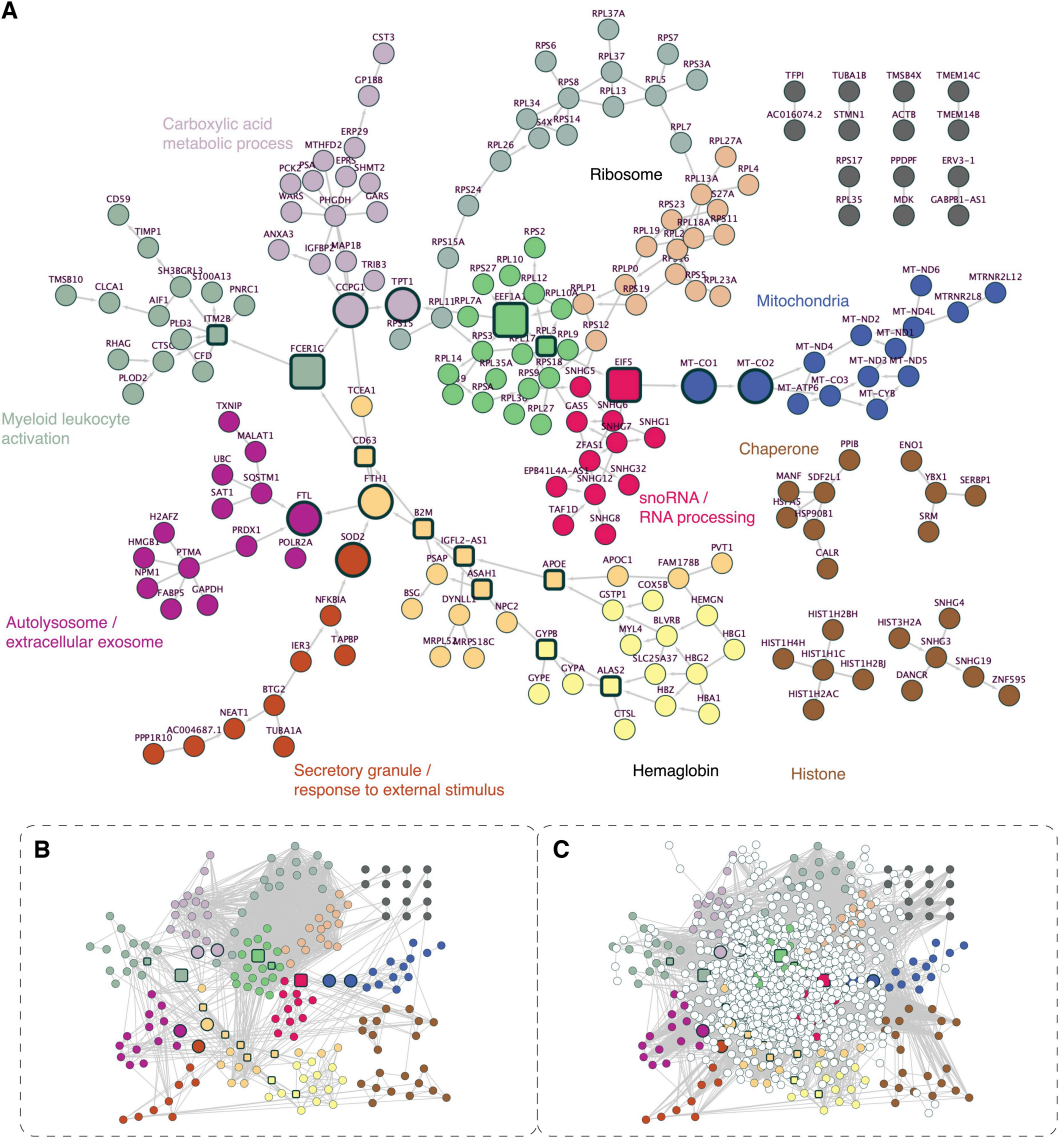
The LatentDAG network. (A) Visualization of the LatentDAG network with a force-directed layout. The color shows the community detection result based on the Leiden algorithm. Annotations of a few clusters are shown. Manual adjustments of the color and position for the seven isolated pathways and four small modules (bottom right) were applied for better visualization. Directions of the edges were shown if the directions were kept after converting the DAG to a CPDAG (see Section 2). We identified a list of genes wherein the removal of one gene resulted in the separation of a large group of genes from the network (see Section 2), and another list of genes with high betweenness. These two gene sets were highlighted by a wider node border. The separator genes were further highlighted with a larger node size and the genes with high betweenness were shown in square. (B) The co-expression network of the same genes in the same node layout. Edges with co-expression value greater than 0.5 were shown. (C) The full co-expression network of all genes. The network was first layouted with the force-directed algorithm and then aligned the genes in panel (A) to their layout in panel (A).

GO and KEGG pathway enrichment analysis was conducted using g: Profiler (Raudvere *et al.* 2019). Visualization of the LatentDAG was performed using Cytoscape (Shannon *et al.* 2003) with the prefuse forced-direct layout. Leiden community detection was carried out using the Python package leidenalg (https://github.com/vtraag/leidenalg). For better visualization, we manually grouped the seven isolated pathways into one cluster, and the four small modules into one cluster. We also adjusted the position of the nodes from these two clusters to make the figure more compact. The DAG was converted to a CPDAG using the Python package causaldag. The DAG edges with directions kept in the CPDAG were shown with the directions in Fig. 1. Other edges were shown as undirected.

For separator genes, we iterated all the genes in the network main component and selected the gene if the removal of the gene resulted in at least two separated components and each component having at least seven genes. Partial correlations between genes in and outside the gene module were calculated using Python package pingouin (Vallat 2018). We used RcisTarget (Aibar *et al.* 2017) to identify TFs that regulate each gene module.

We define the gene knockout response between two genes (e.g. YBX1 and ENO1) as the following: *Z*(*A*|*B*) = Expression(*A*|*B*) – Mean(*A*|Ctrl)/Std(*A*|Ctrl). Essentially, the response of gene A under gene *B* knockout is the expression value of gene *A* under gene *B* knockout normalized by the gene *A* expression under a control condition (without any gene knockout).

All kernel density plots were generated using the kdeplot function from the seaborn package (Waskom 2021) with default parameters.

Prediction of the direct physical interaction between YBX1 and ENO1 proteins was carried out using PEPPI (Bell *et al.* 2022).

A gene pair with its genes located at the start and end of length-two edges from a network was considered an overlap if an edge in the LatentDAG connected these two genes. We first calculated this value based on combining the ChIP GRN (from ENCODE) and BioGRID PPI network. We then sampled an Erdős–Rényi random network with the same size as the BioGRID PPI network. We calculated the number of overlaps for the network combining the ChIP GRN and this random network. The process was repeated 50 times for 50 random networks to retrieve the mean and standard deviation of the number of overlaps. One-sided *P*-value was calculated by the *Z*-score using a normal distribution with the sampled mean and standard deviation.

### 2.4 The prediction models

We first randomly separated all the genes into 70% training, 20% validating, and 10% testing sets. This process was repeated five times with different random seeds.

We train the MLP models [with Python package scikit-learn (Pedregosa *et al.* 2011)] using the training and validating set and reported the mean-squared error (MSE) on the testing set. We used same three hidden layers and same search range for the number of nodes as the GCN model below.

We then construct the GNN model with the model structure shown in Fig. 5B. We used pytorch for the overall structure and used the GCNconv function from the pyg package (Fey and Lenssen 2019) with default parameters for graph convolution. Hyperparameters included the learning rate, number of epochs for training, and number of nodes for the three hidden layers. We used the training set to train the model and select the hyperparameters based on the MSE performance on the validating set. This process was done using the optuna package (Akiba *et al.* 2019). After the determination of the hyperparameters, we trained the model using the training and validating set and reported the MSE or Pearson’s correlation on the testing set. We ran the whole process (including the hyperparameter tunning and final training) 10 times with different random initializations on each train-test split, resulting in a total of 50 runs, to obtain the mean and standard error of the performance.

Notice that for some input networks, not all the nodes were included in the networks. The graph convolution layer would effectively function as a fully connected layer for these nodes. In order to highlight the Bayesian networks, we first identified the subset of genes that existed in the LatentDAG network. We use the train_test_split function from the sklearn package with the “stratify” parameter setting to this gene subset to perform a stratified split. We then performed the same whole process with this new train-validate-test set. All results in the article were based on this set. Additionally, we reported the performance on the testing set but limited to those genes that existed in the LatentDAG as well (Supplementary Fig. S8).

For deeper message-passing in the network, we extend the model by adding another one or two GCNConv + Relu activation layers before the fully connected layers. For other convolution methods, we replaced the GCNConv function with the GATv2Conv function from the pyg package (with default parameters) to incorporate attention into the model. Alternatively, we replaced the two GCNConv layers with a single SGConv function from the pyg package. With parameter *K* = 2, this simple graph convolution represented two layers of convolution but omitted the activation functions between them. Finally, we replaced the GCNConv function with the SAGEConv function. To use an inductive setting for the GraphSAGE model, we have altered the input graph. When training for hyperparameter searching, the input graph was a subgraph for all the training nodes. When training for final model, the input graph was a subgraph containing all training and validating nodes.

### 2.5 Clustering using graph neural network embedding

To retrieve the embedding, we first selected the model with the smallest MSE from the 50 replications. We extracted the embedding after applying the activation function on the last graph convolution layer. Following the same clustering pipeline in the perturb-seq paper (Replogle *et al.* 2022), we first standardized the data, applied minimum-distortion embedding *(*using python package pymde (Agrawal *et al.* 2021) with parameter embedding_dim = 20, n_neighbors = 7, repulsive_fraction = 5) and used HDBSCAN (McInnes and Healy 2017) *(*using python package hdbscan (McInnes *et al.* 2017) with parameter metric=‘euclidean’, min_cluster_size = 10, min_samples = 10, cluster_selection_method=‘leaf’). We then applied the hdbscan.flat.HDBSCAN_flat function to limit the clusterer to output a desired number of clusters. The Replogle clusters were retrieved directly from the perturb-seq paper. The STRING scores for the gene pairs were retrieved from the STRING database (9606.protein.physical.links.v11.5.txt) (Szklarczyk *et al.* 2019).

### 2.6 Other prediction tasks

Additionally, we collected the gene expression data (TPM) of EBV-transformed lymphocyte cells from the GTEx portal. For all the genes, we calculated their mean expression values across the samples and kept genes that have the mean expression larger than the 95% percentile. Similarly, we calculated the expression standard deviation and kept genes with a standard deviation larger than the 95% percentile. This results in a list of 2139 genes, similar to the scale of the perturb-seq dataset, and they are relatively active and variable.

We collected a series of biological networks from the same data source. For ENCODE ChIP-seq data, we filtered the cell line data to obtain a GM12878-specific network. For co-expression, we built the co-expression from the GTEx dataset itself, as well as the collected total RNA-seq of the GM12878 cell line from the ENCODE portal. We applied the same 0.75 threshold. For the Bayesian network, we ran DAGMA on the GTEx expression in the same way.

We then applied the min-max scaling on the input expression and applied the similar stratified train-validate-test split. The model structure and training process were the same as those in the previous model. We used these GTEx expressions and networks to predict the gene conservation level (phyloP). We downloaded the phyloP score (Pollard *et al.* 2010) from the UCSC genome browser (Navarro Gonzalez *et al.* 2021) (https://hgdownload.soe.ucsc.edu/goldenPath/hg38/phyloP100way/hg38.phyloP100way.bw) and used the average score across the gene region as the score of the gene. In addition, we downloaded the phastCons score (Siepel *et al.* 2005) from the UCSC genome browser (https://hgdownload.soe.ucsc.edu/goldenPath/hg38/phastCons100way/hg38.phastCons100way.bw) and processed it in the same way we did for the phyloP score. We used the same GTEx expression and networks to predict gene phastCons score as well.

We collected the expression levels of the genes without any perturbation (wild type) from the perturb-seq dataset as well. We used the perturb-seq expression (gene expression under perturbations) to predict this wild-type gene expression. The model structure and networks were the same as those in the main representative prediction task.

We collected the gene ontology annotation of each gene from the Ensembl Biomart. We used the perturb-seq expression to predict the number of GO terms annotated with the genes. The model structure and networks were the same as those in the main representative prediction task.

## 3 Results

### 3.1 Bayesian network structure learning from gene expression

To demonstrate the value of the Bayesian network, we applied it in a number of established contexts where biological networks were commonly used. In particular, we first collected a representative expression dataset (Replogle *et al.* 2022) and applied an advanced Bayesian network structure learning algorithm DAGMA (Bello *et al.* 2022) on the expression matrix (see Section 2). We referred to the learned network as LatentDAG (Fig. 1), as we considered it to possess the latent structure of the regulatory relationships between genes. Note that a directed Bayesian network is only one possible structure modeling the joint distribution, among all other possible structures, known as Markov-equivalent cases. For example, *P*(*A*)*P*(*B*|*A*) and *P*(*B*)*P*(*A*|*B*) represent the same distribution, but they are shown as two different networks (two nodes with opposite edge direction). Distinguishing the real causal structure from others remains a challenge in Bayesian network learning. It has been reported that a representative Bayesian network structure learning method NOTEARS mainly determined the edge direction based on the variance, with the node having a smaller variance pointing to the other (Seng *et al.* 2022). Therefore, we used the LatentDAG without direction for further analysis. For visualization in Fig. 1, we built the completed partially directed acyclic graph (CPDAG) from our LatentDAG. The CPDAG removed most edge directions but kept the directions that were consistent across equivalent cases.

As references, we collected and constructed other biological networks including ChIP-seq-based GRNs, PPI networks, and co-expression networks (Fig. 1B and C) from various databases and resources (see Section 2). Notice that the expression dataset for constructing the Bayesian networks, as well as some ENCODE datasets, were collected from the K562 cell line, while most of the other networks originated from a mixture of human samples. Compared to all other networks, the LatentDAG was much smaller in size (Supplementary Fig. S1), with only 234 edges connecting 199 nodes. This was around 400 times sparser than a co-expression network built from the same data. The LatentDAG consisted of one main weakly connected component, four small modules, and seven isolated gene pairs (Fig. 1). We noticed that genes naturally formed several clusters in the graph, and we further confirmed this by applying the Leiden algorithm for community detection (Traag *et al.* 2019) (Fig. 1). As the most outstanding ones, we found three groups enriched for ribosome proteins and several groups related to mitochondria, small nucleolar RNA, chaperone, histone, etc. GO term and KEGG pathway enrichment analysis on the genes (except ribosome and mitochondria genes) showed significant results for “antioxidant activity,” “programmed cell death,” “response to stress,” etc. (Supplementary Fig. S2), suggesting the LatentDAG only captured the genes that were most relevant to the cell states (under gene knockdown stress).

### 3.2 Analysis of the LatentDAG structure illuminated unique properties

**Figure 2.**
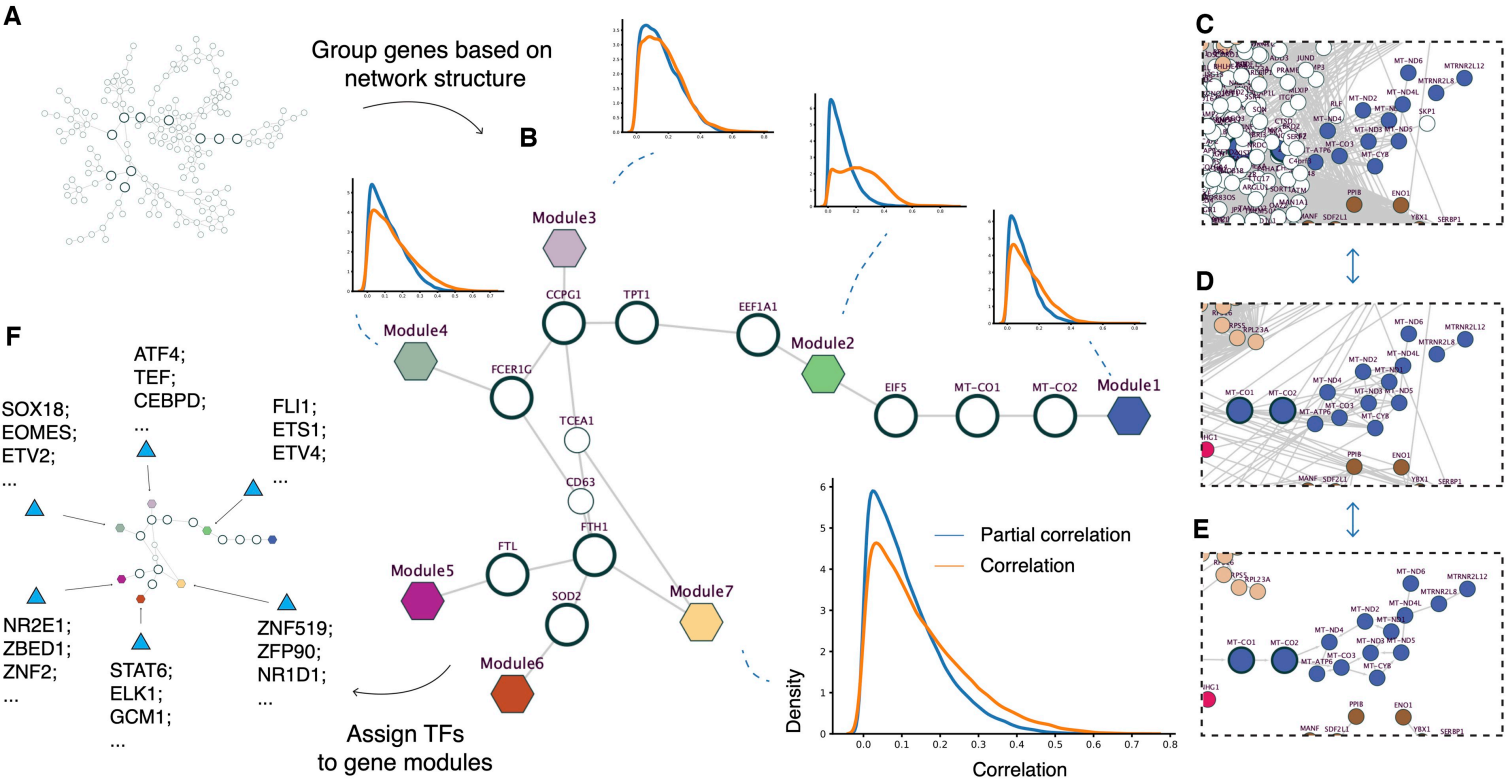
Gene modules based on network structure. (A) The original LatentDAG as in Fig. 1, but only highlighting the separator genes with a wider node border. (B) We identified seven gene modules that could be separated from the network when a single separator gene was removed. For each module, we calculated the correlation between the genes in the module and the genes outside. In addition, we calculated the partial correlation of them conditional on the gene that separated this module from the network (e.g. module1 conditional on MT-CO2 or module2 conditional on EIF5 and EEF1A1). (C) Snapshot of module 1 genes from the full co-expression network. We forced the genes in module 1 to have the same layout as in Fig. 1A. (D) Snapshot of module 1 genes from the co-expression network, removing genes not in LatentDAG. (E) Snapshot of module 1 genes from the LatentDAG. (F) We ran RcisTarget to assign a list of TFs that regulated each gene module. Module 1 was skipped because the mitochondria genes were not included in the annotation database.

According to the definition of Bayesian networks, the network structure indicates the conditional independence between the transcriptomic activities of groups of genes. Particularly, the genes located at bridges connecting large subcomponents should play crucial roles in gene regulation. Therefore, we iterated through all nodes and identified a list of genes where the removal of one gene resulted in a large group of genes separating from the network (see Section 2, Figs 1 and 2A). These genes will be hereafter referred to as the “separator genes.” We then condensed the genes isolated by the separators into several modules (Fig. 2B). In most cases, the correlations between a gene within a module and another gene outside, conditioning on the separator, was closer to zero comparing to regular correlation without conditioning (Fig. 2B).

During the Bayesian network structure learning, genes with low expression and/or low expression variation were largely dropped (Supplementary Fig. S3, white nodes in Fig. 2C). In particular, if we ranked the genes by considering both absolute expression and variance, the LatentDAG genes were highly enriched at the top, with only 11 genes in the top 100 genes not in the LatentDAG and 0 LatentDAG genes in the bottom 1000. Thus, the LatentDAG was greatly enriched in genes that contribute the strongest signal to the co-expression.

As expected, our LatentDAG gene modules had very strong intra-module correlation compared to inter-module ones (permutation test *P* < .0004 compared to random gene sets, or two-sided *t*-test *P* < 3e-12 compared to genes outside the module). Comparing the removed edges in Fig. 2D and E for a given module, the LatentDAG removed many connections to the outside of the module and only left a few intra-module links to summarize the relationships between genes. Therefore, we considered our gene modules as the backbones of important regulatory modules. Finally, by scanning TF motifs in the transcription start sites (TSS) regions of genes with additional predefined motif ranking, RcisTarget (Aibar *et al.* 2017) could identify the TFs that regulated given gene sets, such as gene clusters from co-expression, with high accuracy *(*Santana *et al.* 2024). By applying this approach, we were able to expand the regulatory modules by finding the TFs that regulated the genes in each identified module (Fig. 2F).

Among all our separator genes, *MT-CO1* stood out as a good example to demonstrate regulatory importance. Given the state of *MT-CO1*, the states of mitochondrion genes were conditionally independent on the states of other genes, if the network perfectly satisfied the Bayesian network assumptions. In other words, knowing the states of genes in the cells provided little additional information about the genes in the mitochondria, if we already knew the states of *MT-CO1*. Indeed, we found that the average partial correlation between the mitochondria genes and all other genes conditioning on *MT-CO1* was −0.071, which was not perfectly zero but significantly (two-sided *t*-test, *P* < 6.2e-229) closer to zero than the normal correlation without the *MT-CO1* condition. As the last enzyme in the mitochondrial electron transport chain, MT-CO1 activity roughly reflects the ATP production activity, which is the main function of mitochondria. If we know the ATP production is normal, then the regulation within the mitochondria will not heavily affect or depend on other biological processes happening in the cytoplasm or the cell nucleus.

**Figure 3.**
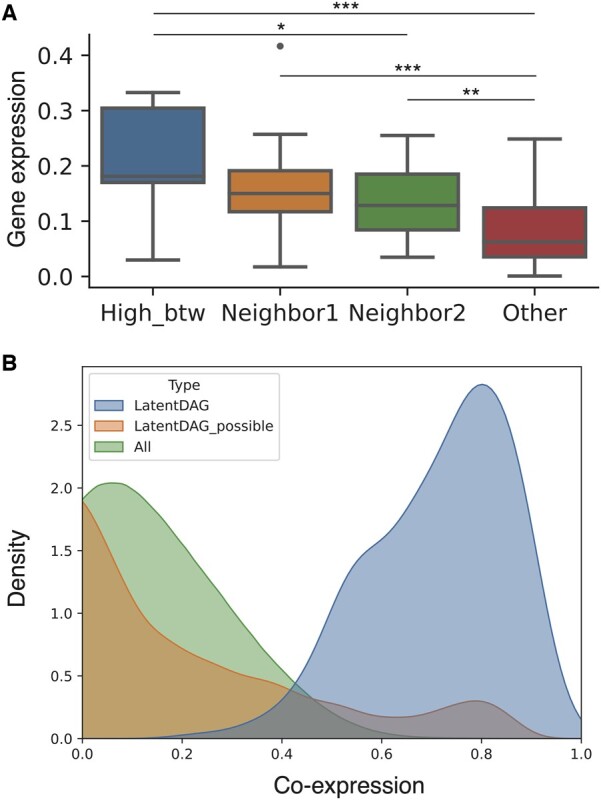
The biological relevance of the LatentDAG nodes and edges. (A) Genes with high betweenness showed higher mean expression levels than genes with low betweenness (all other three groups together, two-sided Welch’s *t*-test, *P* < 2.5e-3). A gradual decrease in the mean expression was observed as we went further away from the high betweenness gene in the network. High_btw: genes with high betweenness; Neighbor1: neighbors of High_btw genes, except those already in High_btw; Neighbor2: neighbors of Neighbor1 genes, except those already in High_btw and Neighbor1; Other: all other genes in the networks but not in the previous three groups. Two-sided Welch’s *t*-test was used. *, **, and *** represented *P*-value smaller than .05, .01, and .001, respectively. (B) The LatentDAG edges overlapped more with high co-expression. LatentDAG: all gene pairs existed as the LatentDAG edges. LatentDAG_possible: all possible gene pairs for genes in the LatentDAG network, except LatentDAG edges. All_pairs: all possible gene pairs for the whole dataset of 2319 genes, except those in the two previous groups.

Besides the separator genes, we identified another list of potentially important “bottleneck” genes based on high betweenness centrality. We found that these bottleneck genes were more expressed than the rest of the genes in the network (Fig. 3A) in our dataset. Furthermore, we examined all first and second neighbors of the bottleneck genes in the network and evaluated their activity by the average absolute expression (see Section 2). The neighbors showed a gradual decrease in the expression level but remained significantly higher than other genes (Fig. 3A). This result suggested that regulations associated with these bottleneck genes may be the important ones relevant to the given cell states.

The fundamental idea of network structure learning in our case was to use gene expression vectors to make a connection between two genes. This resembled co-expression conceptually, and we have found high co-expression within gene modules. Without any constraints, overall, LatentDAG edges overlapped more with the highly correlated gene pairs (Fig. 3B), indicating the potential functional relationships for LatentDAG edges. We then investigated if the LatentDAG exhibited other types of relationships. Around half of the LatentDAG edges overlapped with physical interaction edges in the BioGRID and STRING databases (Supplementary Fig. S1). There were many intersections between the LatentDAG and the co-expression network constructed from the GTEx dataset as well. Surprisingly, the LatentDAG barely had any overlap edges with the ChIP-seq GRNs. Although 88.4% of the LatentDAG genes showed up as TF targets in the ENCODE ChIP-seq GRN, the low level of overlap was largely due to the rare existence of TFs in the LatentDAG. The discrepancy between samples of the data origination contributed to the discrepancy between networks as well. Altogether, these suggested that the LatentDAG might represent heterogeneous relationships, potentially the most functionally relevant and sample-relevant ones.

### 3.3 Example of the LatentDAG representing complicated functional relationships

**Figure 4.**
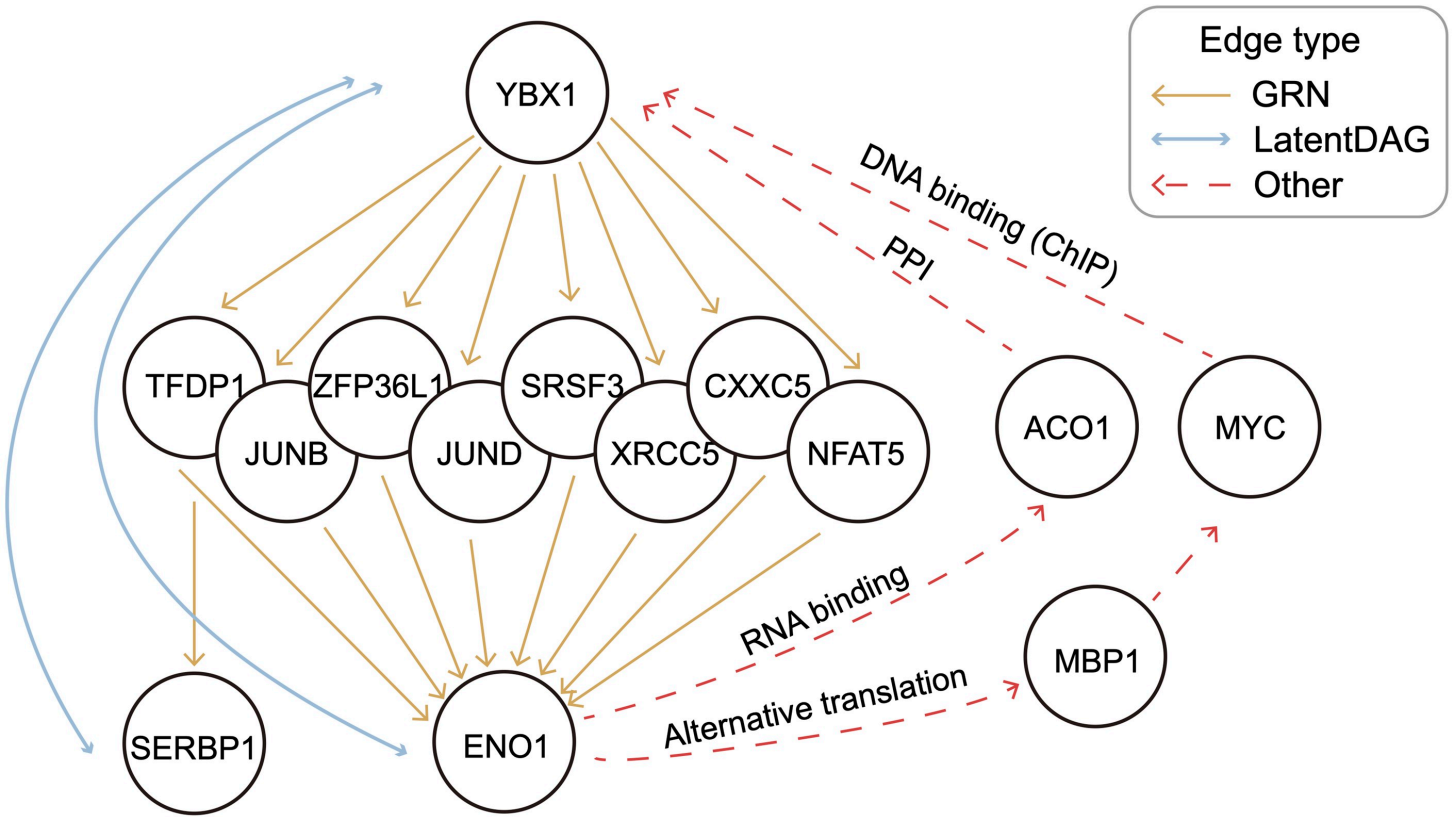
Examples of LatentDAG edges representing co-functions. In ChIP-seq-based GRN, YBX1 regulated several genes in the middle layer and further regulated ENO1 and SERBP1. However, YBX1 and ENO1 or SERBP1 were directly connected in the LatentDAG, showing their co-functional relationship. We also identified various other types of relationships connecting these two genes.

Naively, we expected that LatentDAG could represent direct regulations, such as TF binding to its target genes shown in a ChIP-seq network. However, only three TFs ended up appearing in the LatentDAG, including the well-studied RNA polymerase *POLR2A* and a subunit of the protein phosphatase 1 *PPP1R10*. We therefore tried to further explore the regulation relationships for the last case, *YBX1*, seeking evidence to explain the observation. Y-box binding protein 1 (*YBX1*) was an important TF known to be involved in various processes including DNA repair, mRNA splicing, cancer proliferation, etc. (Capowski *et al.* 2001, Gaudreault *et al.* 2004, Chen *et al.* 2019). Figure 4 shows a small regulation module of YBX1 found in the ChIP-seq GRN and the LatentDAG. Noticeably, there was a three-level hierarchy structure in the GRN. However, the top *YBX1* gene was only directly connected with the bottom genes *ENO1* and *SERBP1* in the LatentDAG, skipping the middle genes. We hypothesized that genes connected by the LatentDAG edges were genes functionally relevant. We then examined the *YBX1–ENO1* gene pair in detail.

The Enolase 1 (*ENO1*) gene has long been known for being related to various types of diseases, including lung cancer and cancer-associated retinopathy (Cappello *et al.* 2009, Pastor *et al.* 2013, Adamus 2017). Particularly, it has been characterized that *ENO1* is overexpressed in triple-negative breast cancer (TNBC) samples (Lai *et al.* 2021). *YBX1* was found to be highly correlated with *ENO1* and it overexpressed in TNBC samples as well (Lai *et al.* 2021). Chen *et al.* (2019) had identified *YBX1* and *ENO1* as two out of three TF markers for breast cancer. Researchers also found that *YBX1* and *ENO1* were the two most significant genes that progressively decreased in expression during the acinar cell development (Hauser *et al.* 2020), providing a potential explanation for the relation between the overexpression of these two genes and breast cancer.

In the dataset, the expression of these two genes had a correlation of 0.746 across all different conditions. We noticed that knocking out *ENO1* significantly reduced the expression of *YBX1* but knocking out *YBX1* only reduced *ENO1* expression marginally (see Section 2). We then tried to find a potential explanation for these results, as we did not observe pathways from *ENO1* to *YBX1* in the GRN. Proteins of these two genes were not predicted to directly interact with each other either (see Section 2). However, *MBP-1*, a product of the alternative translation of *ENO1* genes, could be an interesting lead. The c-myc promoter-binding protein 1 (*MBP-1*) was known to negatively regulate the transcription of the c-myc (Feo *et al.* 2000, Lung *et al.* 2010). Moreover, *YBX1* existed as a target of *MYC* in our ChIP GRN, providing a potential pathway connecting *ENO1* to *YBX1* (Fig. 4). Alternatively, *ENO1* could function as an RNA-binding protein. When binding to the CpG-rich region of the *ACO1* mRNA, it triggers the degradation of the mRNA (Zhang *et al.* 2022). ACO1 protein was known to physically interact with YBX1 (Wang *et al.* 2015), which could serve as a second potential pathway to explain our observation (Fig. 4). Together, these heterogenous relationships were largely summarized by the final expression of the *ENO1* and *YBX1* and were represented by the LatentDAG. Finally, as an additional support of this trend in general, we focus on the length-two edges that represent long-distance relationships. A gene pair with its genes located at the start and end of a length-two edge from a network was considered an overlap if an edge in the LatentDAG connected these two genes. Despite the lack of overlap between the LatentDAG and ChIP network, we observed a significant increase (*P* < .038, see Section 2) of overlaps as we added the PPI relationships on top of the ChIP network.

Another example showing the potential function relationships was between the *FTL*–*PRDX1* pair and the *FTH1*–*SOD2* pair in the LatentDAG. In response to oxidation stress, superoxide was first converted to peroxide by SOD2, then further converted to nontoxic water by GPX1, PRDX1, and other proteins (Kim *et al.* 2017). These reactions required iron to provide and accept electrons. The amount of free iron in the cells was controlled by ferritin (Baraibar *et al.* 2010), a protein constituted of FTL, FTH1, and other peptides. Both the *FTL–PRDX1* and *FTH1–SOD2* pairs had relatively low expression correlations compared to other LatentDAG edges (0.24 and 0.33, respectively). However, all these proteins were closely related to each other in the same reaction pathways that were active under stress.

### 3.4 Quantitative benchmark of the LatentDAG

**Figure 5.**
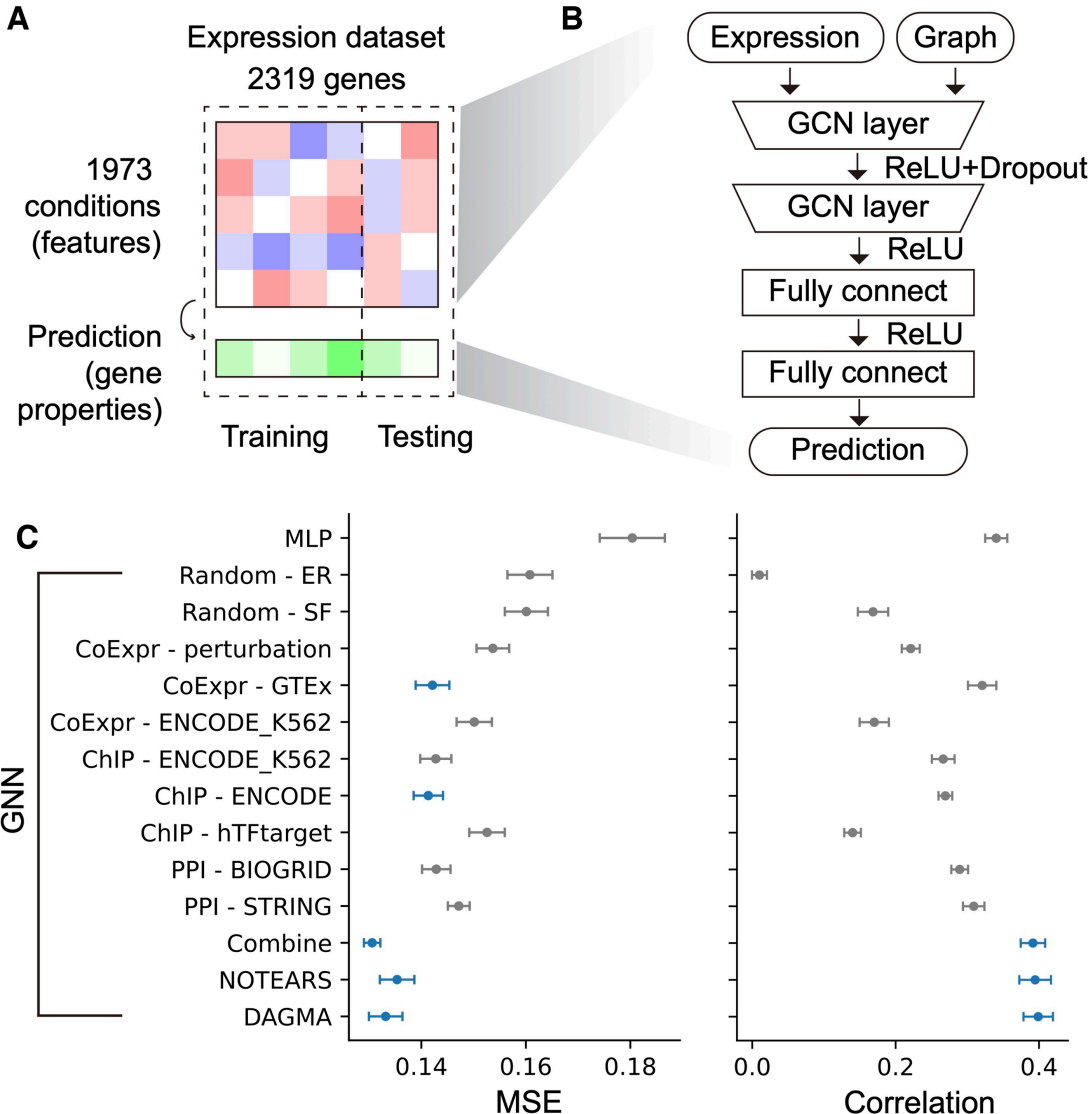
GNN model. (A) Data used in the representative prediction task. Given the expression of a list of genes across different conditions, we used these conditions as features and made predictions of gene properties, such as the conservation level of the genes. We randomly split the expression dataset into training and testing sets to train and validate the models. (B) The neural network model structure for the prediction. GCN: graph convolution network layer. (C) Performance of the different models in terms of MSE and Pearson correlation. Error bars show the standard error from 5 different train-test splits and 10 random model initiations for each split, a total of 50 runs. GNN: GNN with the specified network as part of the input as shown in panel (B); MLP: multi-layer perceptron; Random-ER: random network generated from the Erdős–Rényi model; Random-SF: random scale-free network; ChIP-hTFtarget: network based on ChIP-seq from the hTFtarget database; ChIP-ENCODE: network based on ChIP-seq from all human cell lines on the ENCODE portal; ChIP-ENCODE_K562: network based on ChIP-seq from only K562 cell line on the ENCODE portal; CoExpr-perturbation: co-expression network calculated from the expression data directly; CoExpr-GTEx: co-expression network based on GTEx whole blood data; CoExpr-ENCODE_K562: co-expression network from the expression data from K562 cell line on the ENCODE portal; Combine: combination of ChIP-ENCODE, CoExpr-GTEx and BIOGRID. Gray color indicated that result was statistically significantly (two-sided Welch’s *t*-test, *P* < .05) different to the result of DAGMA, while blue color indicated insignificance.

**Figure 6.**
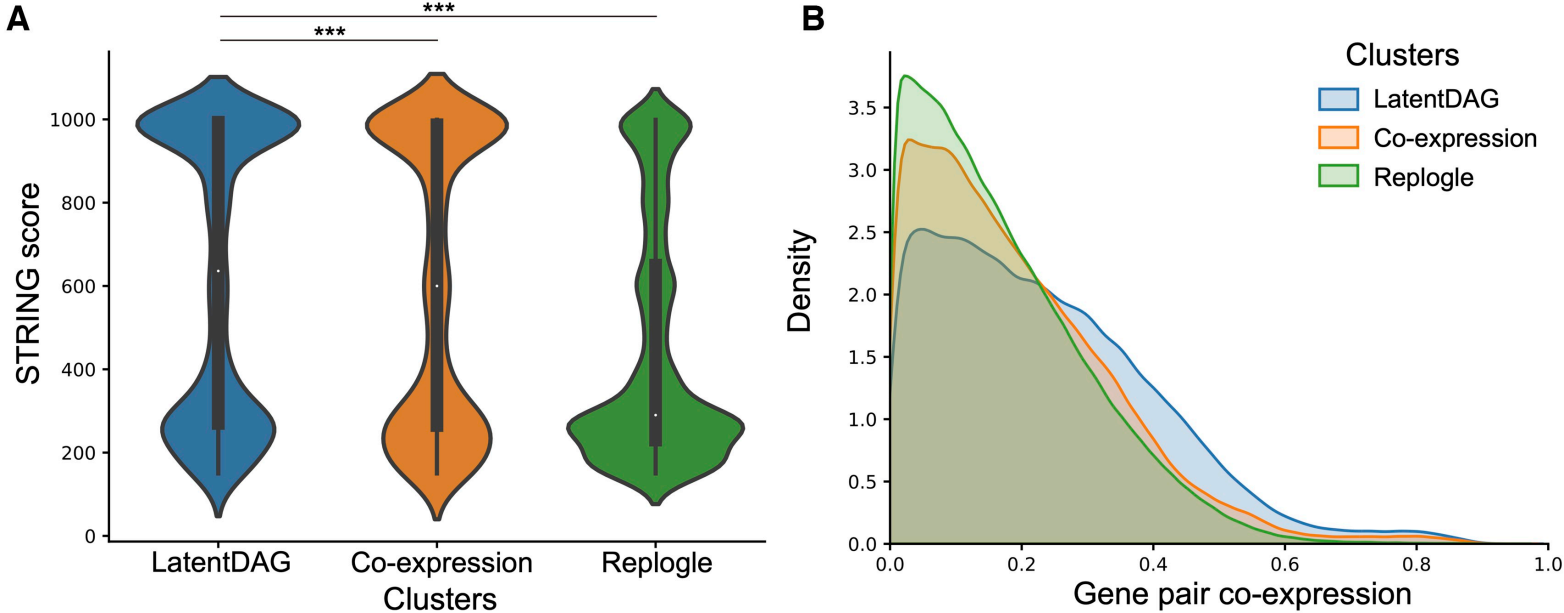
Comparison between LatentDAG clusters and other clusters. We considered all possible gene pairs within each of the clusters and assigned the STRING score (A) or expression correlations (B) for these pairs. LatentDAG clusters and co-expression clusters were generated based on the embedding in the GNN + LatentDAG model and GNN + co-expression model, respectively. The Replogle clusters were calculated based on the expression directly. We observed an enrichment of edges with high STRING scores and high co-expression in the LatentDAG clusters compared to the other clusters.

To evaluate the value of the Bayesian network in the sense of a quantitative benchmark, we employed a GNN model (Fig. 5), as it could efficiently take advantage of better network information and provide easily evaluable statistical metrics. Specifically, we compared the performance of a model using the LatentDAG to one using biological networks in a series of different tasks, including the prediction of (i) gene conservation level (Fig. 5C), (ii) number of functional annotations (Supplementary Fig. S4), and (iii) the expression in different conditions (Supplementary Fig. S5). We also applied the networks to facilitate gene clustering (Fig. 6). To bolster the generality of our conclusions, we evaluated other Bayesian networks besides the one generated by DAGMA. In particular, we ran NOTEARS on the same dataset. Furthermore, we evaluated this approach on different benchmark datasets, i.e. we also determined results on the well-known GTEx expression data (see Section 2) which gives rise to new Bayesian networks. Similar tasks were conducted, such as using GTEx expression and its corresponding network to predict gene conservation level measured by phyloP or phastCons score (Supplementary Fig. S6). Overall, the Bayesian networks showed superior or similar performance when compared to common biological networks. We presented more details of two representative tasks in the following sections.

### 3.5 Representative task one: predicting gene conservation level from expression and LatentDAG

Given the gene expression values under different circumstances, we aimed to predict the conservation level of the genes (Fig. 5A, see Section 2). We randomly split all the genes into training and testing sets and reported the prediction performance as the MSE and Pearson correlation on the testing set. The process was repeated multiple times to achieve robust measurement (see Section 2). We used a GNN model to incorporate the relationships between genes (Fig. 5B). For comparison, we applied a multilayer perceptron model with similar structure and hyperparameter searching range. Surprisingly, the GNN models with some biological networks did not perform as well as MLP in terms of correlation (Fig. 5C), potentially due to the input network quality. We then used the LatentDAG or the NOTEARS network as the graph input. Noticeably, the GNN models with these Bayesian networks outperformed other biological networks, with the DAGMA having the smallest mean MSE and the NOTEARS network having comparable results (Fig. 5C). Significant improvement was also observed using the Pearson correlations (Fig. 2C, Supplementary Fig. S7) as the measurement. Finally, measurements focusing on only the subset of genes highlighted by the LatentDAG network have comparable results between using LatentDAG and other networks (Supplementary Fig. S8).

The relatively larger error in the GNN models using biological networks than those using Bayesian networks might be due to insufficient message passing, as the biological networks were usually complicated. Therefore, we extended the maximum message-passing distance from two hops to three or four hops by introducing more graph convolution layers. Besides, we tested different graph convolution methods, including one using the attention mechanism and another one with simplified graph convolution, to explore if model complexity affected the result. Finally, a LatentDAG relationship might be a summary of heterogeneous relationships. Therefore, we wonder if combining multiple biological networks could help achieve a performance comparable to that of the LatentDAG. To do this, we combined ChIP-seq-based GRN, BioGRID PPI, and co-expression networks to form a heterogeneous network. Neither deep message passing (Supplementary Fig. S9) nor different convolution methods (Supplementary Fig. S10) provided consistent improvement, potentially due to over-smoothing for deep message passing or other limitations rooted in the original network quality. Combining multiple biological networks resulted in decent performance (Fig. 5C) which was similar to that of the DAGMA. However, such a combined network required much more data to build and was highly inter-connected which may limit further interpretation on the network.

To investigate if variation in the graph size (number of edges and nodes) impacts the results, we constructed a series of networks by varying the threshold when defining the co-expression network. We did not find a monotonic trend, as the results from a larger co-expression network with a threshold of 0.2 was similar to the result from a smaller network with a 0.5 threshold (Supplementary Fig. S11). Using a larger “chimera” network by adding lowly co-expressed edges to the Bayesian network also deteriorates the model (Supplementary Fig. S12). However, adding relatively highly co-expressed edges minimized such impact (Supplementary Fig. S12).

Similar to the representative task above, we applied the workflow on the GTEx expression dataset (from EBV-transformed lymphocytes) to predict gene conservation levels as well (see Section 2). We collected similar biological networks (but limited to the GM12878 cell line when possible) and used the same model structure (Fig. 5B). To further test the generalizability, we tested both phyloP score and phastCons score for the gene conservation level measurement. The model with Bayesian networks again achieved decent performance (Supplementary Fig. S6) with a comparable MSE but higher correlation, compared to models in combination with biological networks originating from the GM12878 cell line.

### 3.6 Representative task two: gene clustering by using the embedding from GNN with LatentDAG

Here, we illustrated another example of using Bayesian network and GNN models. One advantage of the GNN model was that it learned an embedding for each of the nodes. Compared to the original node features (raw expression), the embedding for each node intrinsically summarized the information from its neighbors on top of its own and thus provided more information. In the original paper where the expression data were generated, Replogle and colleagues identified 38 gene clusters using the expression profile (Replogle *et al.* 2022), hereafter referred to as Replogle clusters. We applied the same clustering pipeline on the embedding from the GNN model with the LatentDAG and further limited the result to form 38 clusters as well (see Section 2). Due to the nature of the clustering algorithm HDBSCAN, 377 genes could not be clustered, fewer than 1133 genes in the original analysis. Similarly, we performed the clustering on the embedding from the GNN model with the data-derived co-expression network. We referred to these two as the LatentDAG clusters and co-expression clusters. We then considered all possible gene pairs within each cluster. For pairs within the LatentDAG clusters, their average STRING score, which measured the confidence level of two genes having a connection, was significantly larger than those within co-expression clusters (two-sided Welch’s *t*-test, *P* < 2.0e-18) or the Replogle clusters (two-sided Welch’s *t*-test, *P* < 1.9e-205) (Fig. 6A). Similarly, they also showed higher co-expression levels than pairs from Replogle clusters (Fig. 6B).

## 4 Discussion

Gene regulation analysis has always been a fundamental field in biomedical studies. To date, around 19 395 protein-coding genes and more than 89k protein-coding transcripts have been found in humans (GENCODE v45) (Frankish *et al.* 2019), but understanding how genes function individually and collectively remains a huge challenge. The complexity rooted in the exponential possible combination between genes puts a tremendous barrier in front of many traditional analyses. Graph-based approaches have often been used to study these complicated biological regulation networks.

Due to its ability to handle complex graph-structured data, the GNN, a powerful type of machine learning model, has gained significant attention in recent years. Unlike traditional neural networks that operate on data such as images and texts, GNNs can effectively capture the relationships and interactions among nodes in a graph. This unique capability makes GNNs ideal for a wide range of tasks, including social network analysis, drug discovery, and recommendation systems, among others (Wu *et al.* 2020).

When applying GNN models, the quality of the network is undoubtedly vital. Unfortunately, this is a concerning problem in many biological networks. For example, ChIP-seq is well known to suffer from high noise, largely due to the unspecific binding of the antibody (Xu *et al.* 2021). Therefore, when constructing a GRN to represent TF–target relationships, it would likely find more targets than it should because of the spurious binding. Moreover, in typical GNN analysis, the network is assumed to be static. However, this is not the case for a biological system, where network rewiring happens a lot between different conditions (Li and Johnson 2010). Dynamic GNN models have been proposed (Lin *et al.* 2022), but such biological data are not always available. Therefore, the concordance between the network and the observed data (node features) remains crucial in most cases. A typical type of network that has a higher level of concordance is the co-expression network built from an observed RNA-seq dataset. We showed that such a network indeed performed better than other biological networks in some cases (see, e.g. “CoExpr-perturbation” in Supplementary Fig. S9). Moreover, inferring only the most active and the most important relationship directly from the data might be able to further alleviate these problems.

Network structure learning, particularly Bayesian network learning, has been a longstanding problem of study in the field of statistics. Identifying a network structure that explains the data while subjecting it to the DAG constraint is an NP-hard problem (Chickering 1996). However, by connecting the underlying meaning of the matrix exponential to the DAG constraint, Zheng *et al.* successfully converted the combinatorial problem into a numerical problem that could be easily optimized. Inspired by this, many other methods have been proposed, and we applied one of the newest tools, DAGMA. To show that our result is not tool-specific, we performed our main analysis with the foundational tool NOTEARS as well. A total of 92.3% of the genes in the NOTEARS predicted network existed in the LatentDAG, and prediction performance with the GNN models was comparable between these two. Both were better than those using biological networks from other sources (Fig. 5C).

It is worth noting that despite these methods being called “causal learning” in many papers, we remained cautious in this study when interpreting the network. As mentioned, it was challenging to distinguish between a real causal structure and equivalent cases. An additional issue of the NOTEARS was that it was an estimation without rigorous tests of the independence assumptions. In our analysis, we chose the network from DAGMA as the representative, as this new tool was reported to be more accurate than NOTEARS. Additionally, for network visualization, we converted the DAG to the CPDAG that could represent the equivalent cases of a DAG (Fig. 1). Finally, we did not perform reasoning, as it required accurate assignment of local distribution on each node and complicated derivations to represent the target in terms of the conditional probabilities. We only used the Bayesian network as a latent structure that could help improve other analyses, such as building a GNN model. Despite saying these, we could still obtain a few insights from the structure, as shown in the MT-CO1 example. Structure learning, including Bayesian network structure learning, has been known as a technique to improve GNN models in general, and we demonstrated its capability for biological networks as well. Moreover, we showed that this improvement is not only technical but also biologically reasonable, as we observed overlaps between the LatentDAG and biological relationships in various aspects.

We observed that the LatentDAG only includes a small fraction of genes in the network. This could be attributed to a few technical reasons. The expression matrix only contains a list of the genes that were “highly variable” according to the authors of the original paper. Besides, the Bayesian network was known to be able to model the joint probability of all variables with fewer values. Finally, we have added the L1 regularization penalty to the loss, as we do not expect genes to regulate all other genes. On the other hands, a few biological factors could also play a role. Some genes were not captured by the Bayesian network, suggesting they did not strongly co-variate with other genes in our dataset. Indeed, we found that the genes not included in the network had lower absolute expression values and lower expression variation, and found high enrichment of LatentDAG genes in the top 100 genes ranked by both expression and variation. Additional analyses of varying the co-expression threshold and adding co-expression edges to LatentDAG showed that the number of nodes and edges did not directly impact the results. Despite having a relatively small number of genes, we identified a few gene modules that were relatively independent when conditional on a few separator genes, which was different from co-expression clusters that were denser and more interconnected. The genes in each module represented the backbones of regulatory modules, and we were able to replenish these modules by finding and assigning the TFs that regulate each group of genes.

In summary, we inferred a relatively small network directly from the observational gene expression data by applying Bayesian network structure learning methods. We demonstrate that it performed better than other biological networks in a series of GNN-based tasks, including prediction of gene conservation and clustering of genes using GNN embedding. A closer examination of the network highlighted its unique properties and provided interesting genes and interactions that were functionally relevant and warranted further studies.

## Supplementary Material

btae463_Supplementary_Data
