# Supplementary material for: Representing core gene expression activity relationships using the latent structure implicit in Bayesian networks
Source: Bioinformatics. 2024 Jul 25;40(8):btae463. doi: 10.1093/bioinformatics/btae463 (PMC11316617; doi:10.1093/bioinformatics/btae463)
Supplement: btae463_Supplementary_Data [file btae463_supplementary_data.pdf]

Network	# edges	# nodes	# overlap edges
LatentDAG	234	199	-
ChIP_hTFtarget	23898	2171	1
ChIP_ENCODE_Human	59559	2226	1
ChIP_ENCODE_K562	28088	2082	1
CoExp_data	92930	1523	218
CoExp_GTEx	382852	2024	92
CoExp_ENCODE_K562	638258	1907	61
BIOGRID	66579	2209	59
STRING	119776	2128	92
Combine	491041	2311	110

Figure S1. Number of edges and nodes in each biological network. The last column showed the number of LatentDAG edges that existed in other networks.

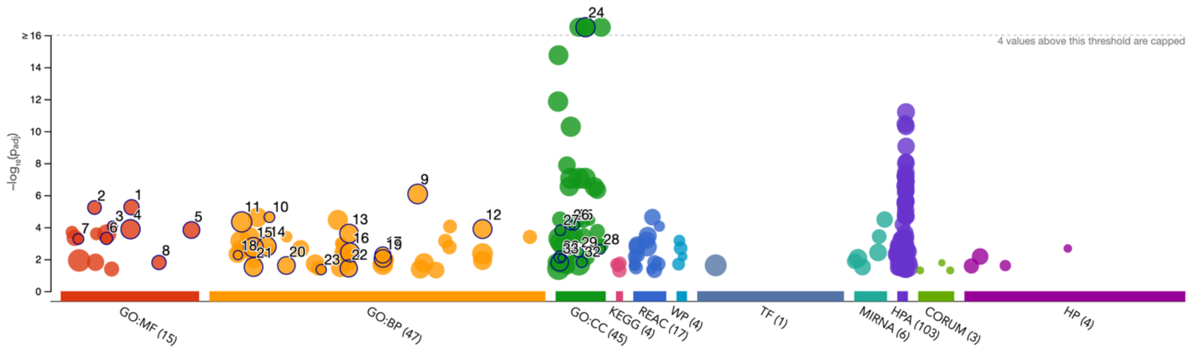

ID	Source	Term ID	Term Name	p_{adj} (query_...
1	GO:MF	GO:0043177	organic acid binding	5.668×10^{-6}
2	GO:MF	GO:0016209	antioxidant activity	5.851×10^{-6}
3	GO:MF	GO:0031720	haptoglobin binding	8.471×10^{-5}
4	GO:MF	GO:0042802	identical protein binding	1.338×10^{-4}
5	GO:MF	GO:0140678	molecular function inhibitor activity	1.487×10^{-4}
6	GO:MF	GO:0019825	oxygen binding	5.007×10^{-4}
7	GO:MF	GO:0005344	oxygen carrier activity	5.399×10^{-4}
8	GO:MF	GO:0051082	unfolded protein binding	1.596×10^{-2}
9	GO:BP	GO:0070887	cellular response to chemical stimulus	8.349×10^{-7}
10	GO:BP	GO:0015670	carbon dioxide transport	2.341×10^{-5}
11	GO:BP	GO:0006950	response to stress	4.643×10^{-5}
12	GO:BP	GO:1901566	organonitrogen compound biosynthetic process	1.295×10^{-4}
13	GO:BP	GO:0044248	cellular catabolic process	2.495×10^{-4}
14	GO:BP	GO:0012501	programmed cell death	1.590×10^{-3}
15	GO:BP	GO:0009605	response to external stimulus	1.811×10^{-3}
16	GO:BP	GO:0044419	biological process involved in interspecies inte...	3.762×10^{-3}
17	GO:BP	GO:0051235	maintenance of location	5.425×10^{-3}
18	GO:BP	GO:0006564	L-serine biosynthetic process	5.526×10^{-3}
19	GO:BP	GO:0051248	negative regulation of protein metabolic process	8.911×10^{-3}
20	GO:BP	GO:0019725	cellular homeostasis	2.459×10^{-2}
21	GO:BP	GO:0009607	response to biotic stimulus	3.061×10^{-2}
22	GO:BP	GO:0044092	negative regulation of molecular function	3.724×10^{-2}
23	GO:BP	GO:0034975	protein folding in endoplasmic reticulum	4.467×10^{-2}
24	GO:CC	GO:0070062	extracellular exosome	7.073×10^{-18}
25	GO:CC	GO:0034663	endoplasmic reticulum chaperone complex	7.210×10^{-5}
26	GO:CC	GO:0031838	haptoglobin-hemoglobin complex	7.210×10^{-5}
27	GO:CC	GO:0005833	hemoglobin complex	1.549×10^{-4}
28	GO:CC	GO:0170014	ankyrin-1 complex	2.394×10^{-3}
29	GO:CC	GO:0042824	MHC class I peptide loading complex	3.576×10^{-3}
30	GO:CC	GO:0005790	smooth endoplasmic reticulum	7.210×10^{-3}
31	GO:CC	GO:0008043	intracellular ferritin complex	8.373×10^{-3}
32	GO:CC	GO:0044754	autolysosome	1.520×10^{-2}
33	GO:CC	GO:0005764	lysosome	1.549×10^{-2}

**version**	e111_eg58_p18_30541362	g:Profiler
**date**	4/16/2024, 2:29:41 PM	
**organism**	hsapiens	

Figure S2. GO enrichment analysis for the LatentDAG genes using g:Profiler. Genes showed significant enrichment for stress-response-related terms, in expectation of the behavior under the gene knockdown environment.

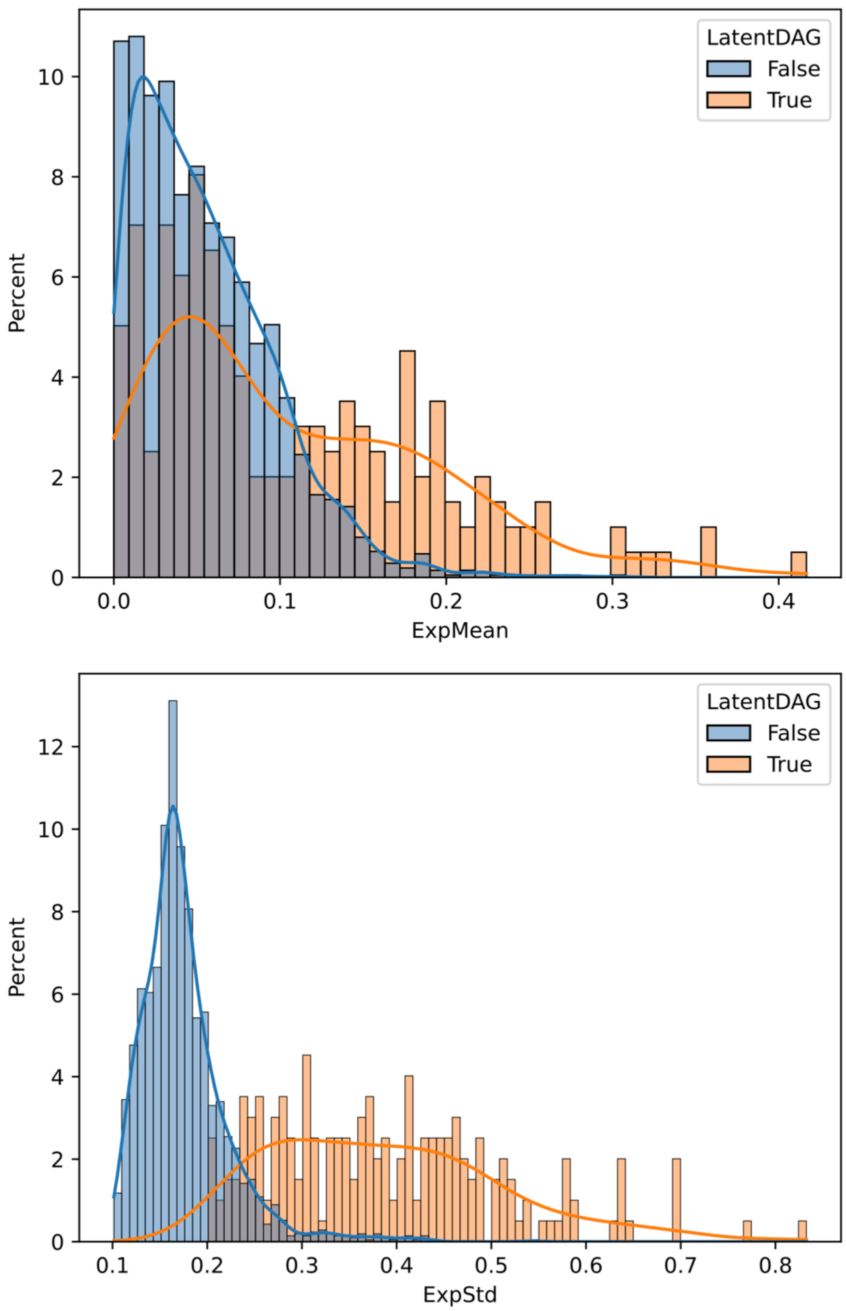

Figure S3. Distribution of the mean of absolute (top) and standard deviation (bottom) of gene expression values for genes in or not in LatentDAG. This suggested the genes in LatentDAG had significantly (two-sided Mann-Whitney U test, $p<6.04e-18$) higher absolute expression values than genes not in the LatentDAG. They also had significantly (two-sided Mann-Whitney U test, $p<5.54e-110$) higher standard deviations.

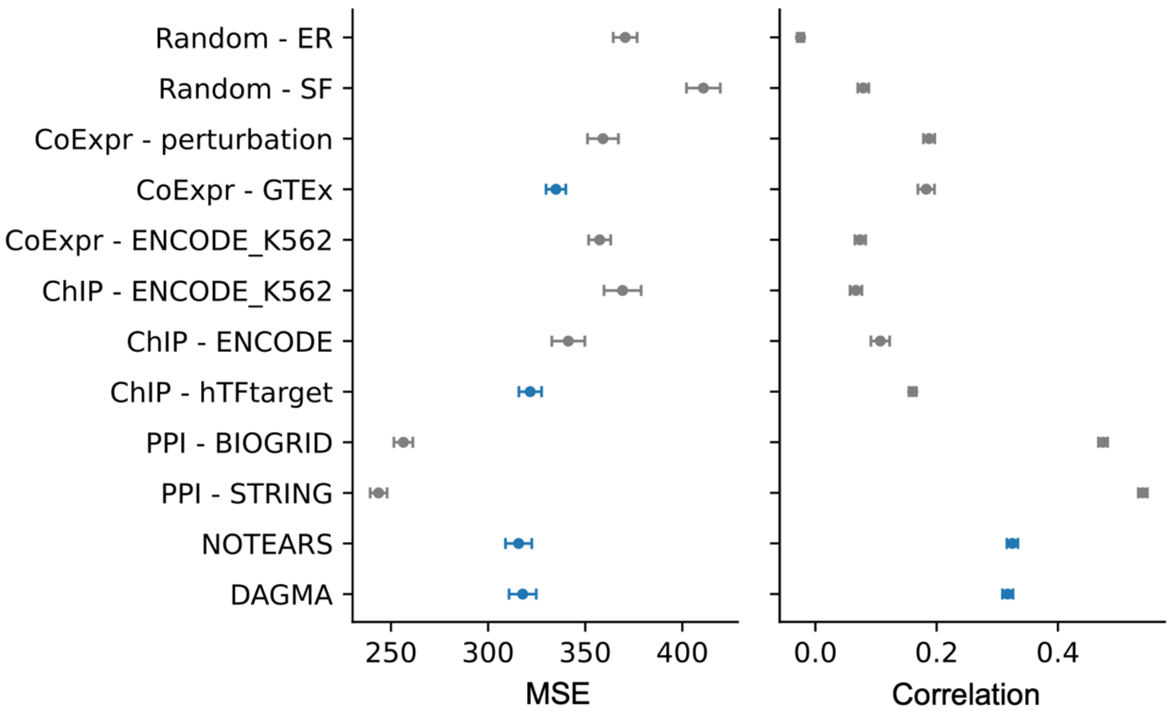

Figure S4. Performance of the GNN models that use gene expression to predict the number of GO terms annotated with the genes. The explanation of each network label was the same as those in the caption of Figure 5C. Error bars showed the standard error from 5 different train-test splits and 10 random model initiations for each split, a total of 50 runs. Results were shown in MSE (left) and Pearson correlation (right). Grey color indicated that result was statistically significantly (two-sided Welch's t-test, $p<0.05$) different to the result of DAGMA, while blue color indicated insignificance.

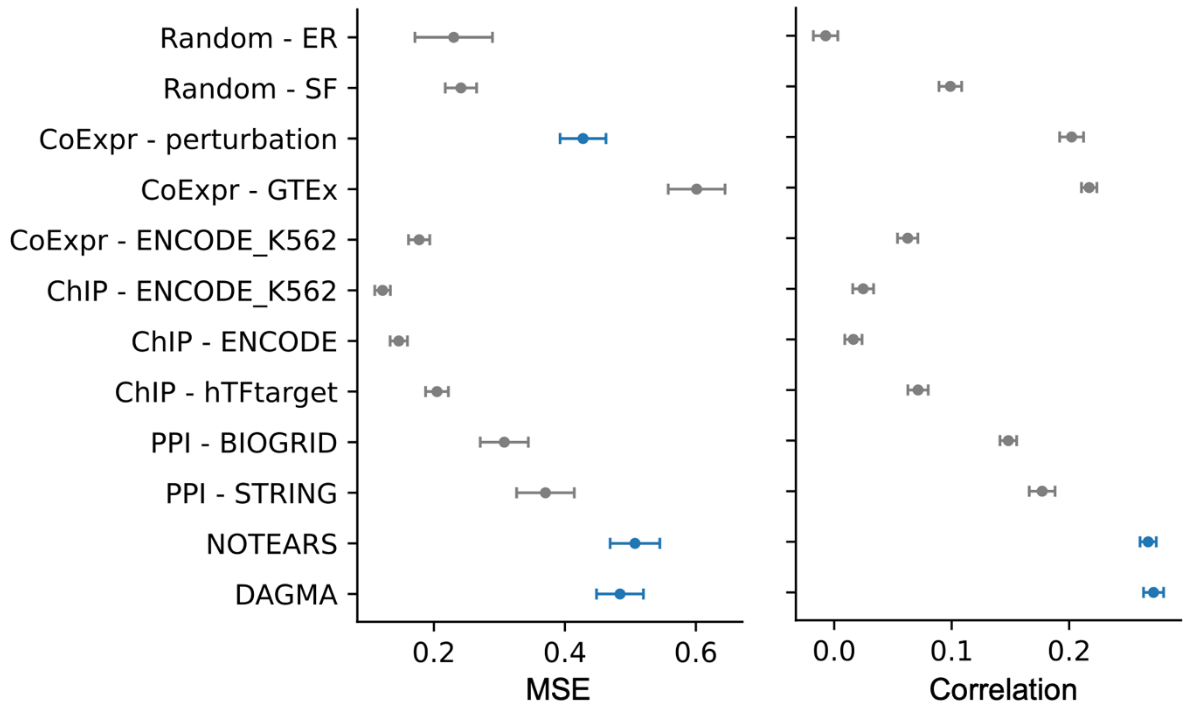

Figure S5. Performance of the GNN models that use expression under perturbations to predict expression in wild type. The explanation of each network label was the same as those in the caption of Figure 5C. Error bars showed the standard error from 5 different train-test splits and 10 random model initiations for each split, a total of 50 runs. Results were shown in MSE (left) and Pearson correlation (right). Grey color indicated that result was statistically significantly (two-sided Welch's t-test, $p<0.05$) different to the result of DAGMA, while blue color indicated insignificance.

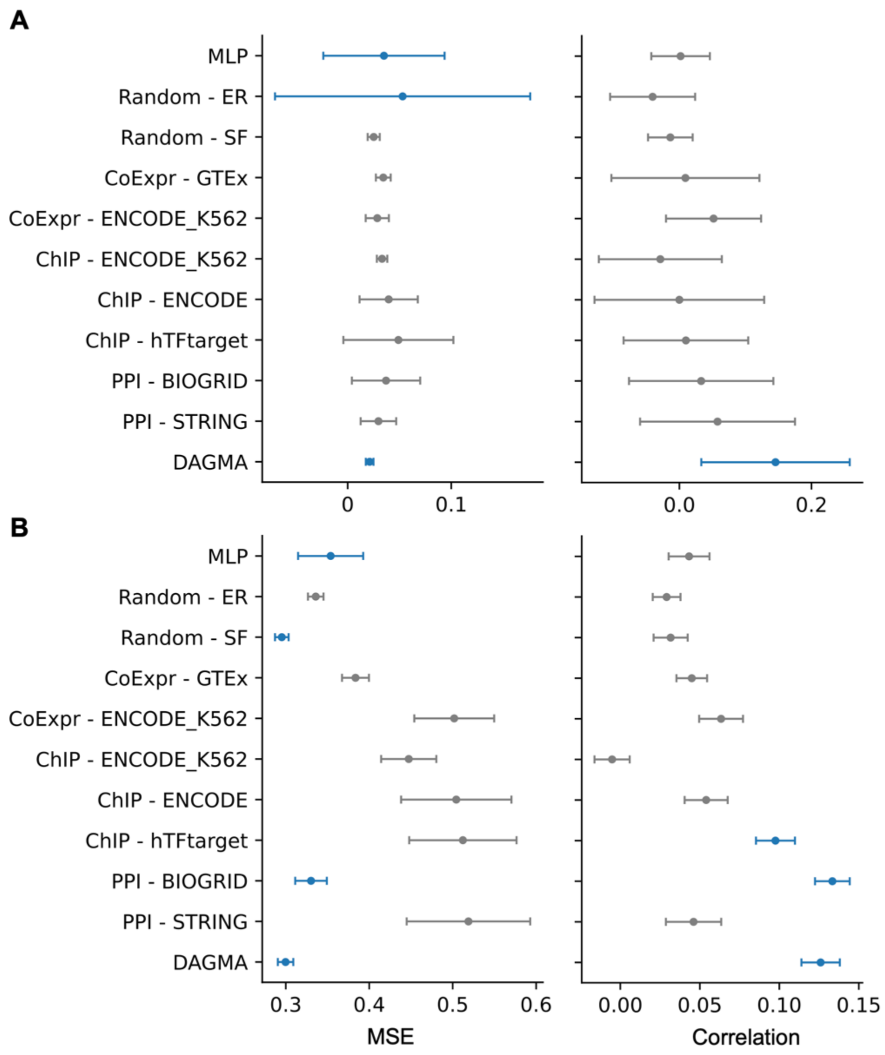

Figure S6. Performance of the GNN models that use GTEx expression to predict gene conservation level. Gene conservation levels were measured by phastCons (panel A) or phyloP(panel B) scores. The explanation of each network label was the same as those in the caption of Figure 5C. Error bars showed the standard error from 5 different train-test splits and 10 random model initiations for each split, a total of 50 runs. Results were shown in MSE (left) and Pearson correlation (right). Grey color indicated that result was statistically significantly (two-sided Welch's t-test, $p<0.05$) different to the result of DAGMA, while blue color indicated insignificance.

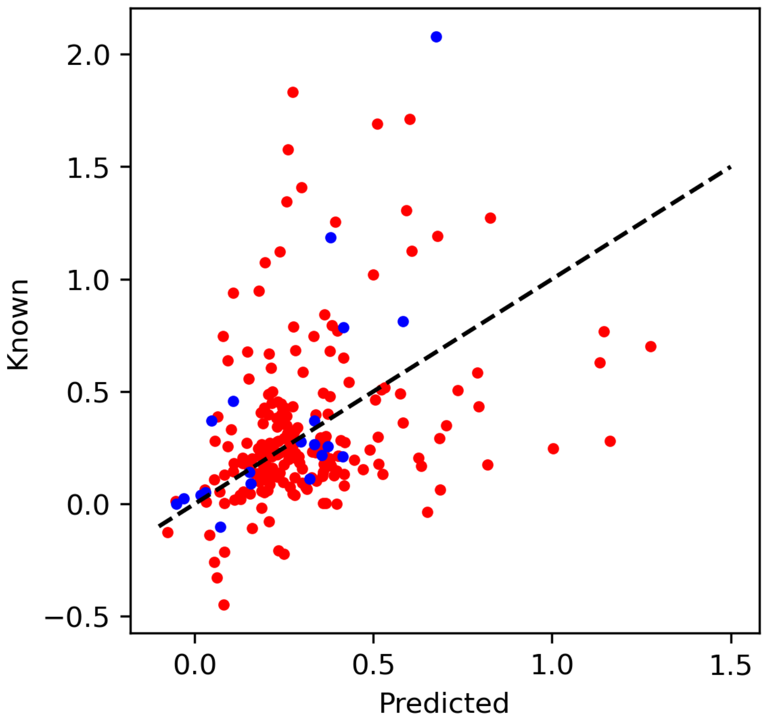

Figure S7. Predicted vs. known result from the GNN model using LatentDAG network. The figure showed the predicted values against the known values in the testing set from one run. The blue dots represent the genes associated with LatentDAG in the testing set, and the red dots represent other genes in the testing set. The black dashed line shows the Y=X line. Pearson correlations of all dots and only the blue dots are 0.345 and 0.727 respectively.

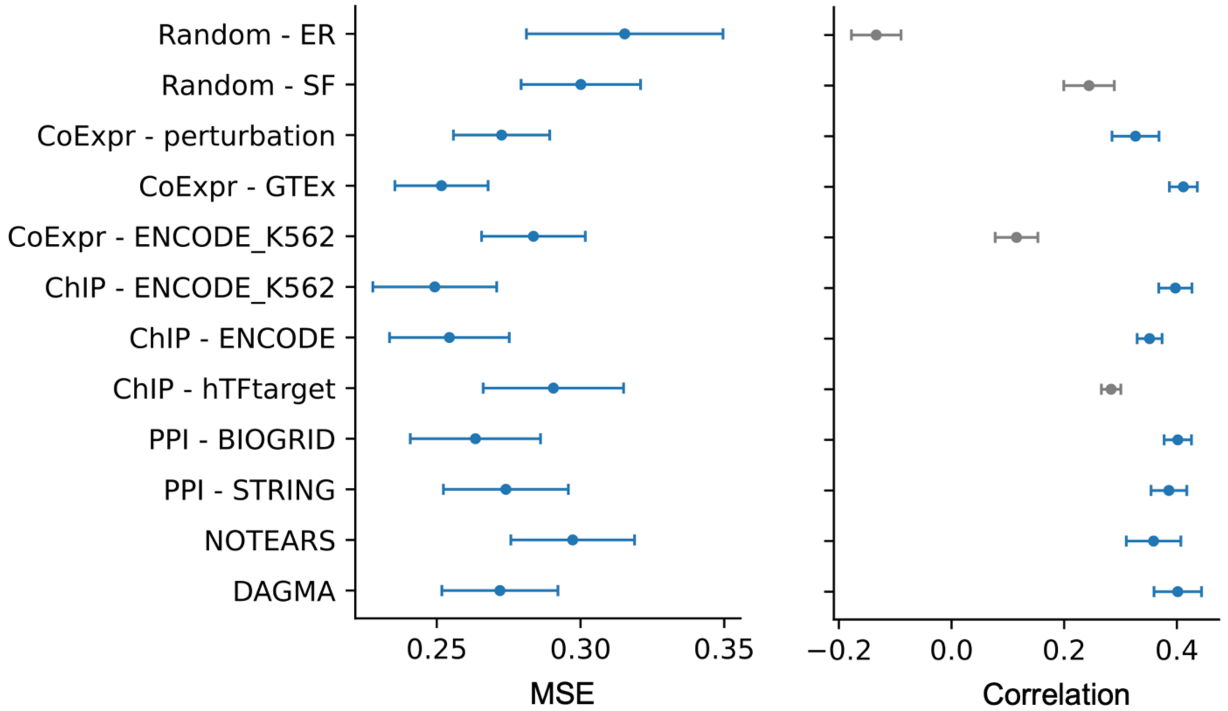

Figure S8. Performance of the representative model but measured on only the genes associated with the LatantDAG. Of all genes, a subset of them was in the LatentDAG network. We used stratified train-test splitting and measured the performance on only the LatentDAG genes in the testing set. The explanation of each network label was the same as those in the caption of Figure 5C. Error bars showed the standard error from 5 different train-test splits and 10 random model initiations for each split, a total of 50 runs. Results were shown in MSE (left) and Pearson correlation (right). Grey color indicated that result was statistically significantly (two-sided Welch's t-test, $p<0.05$) different to the result of DAGMA, while blue color indicated insignificance.

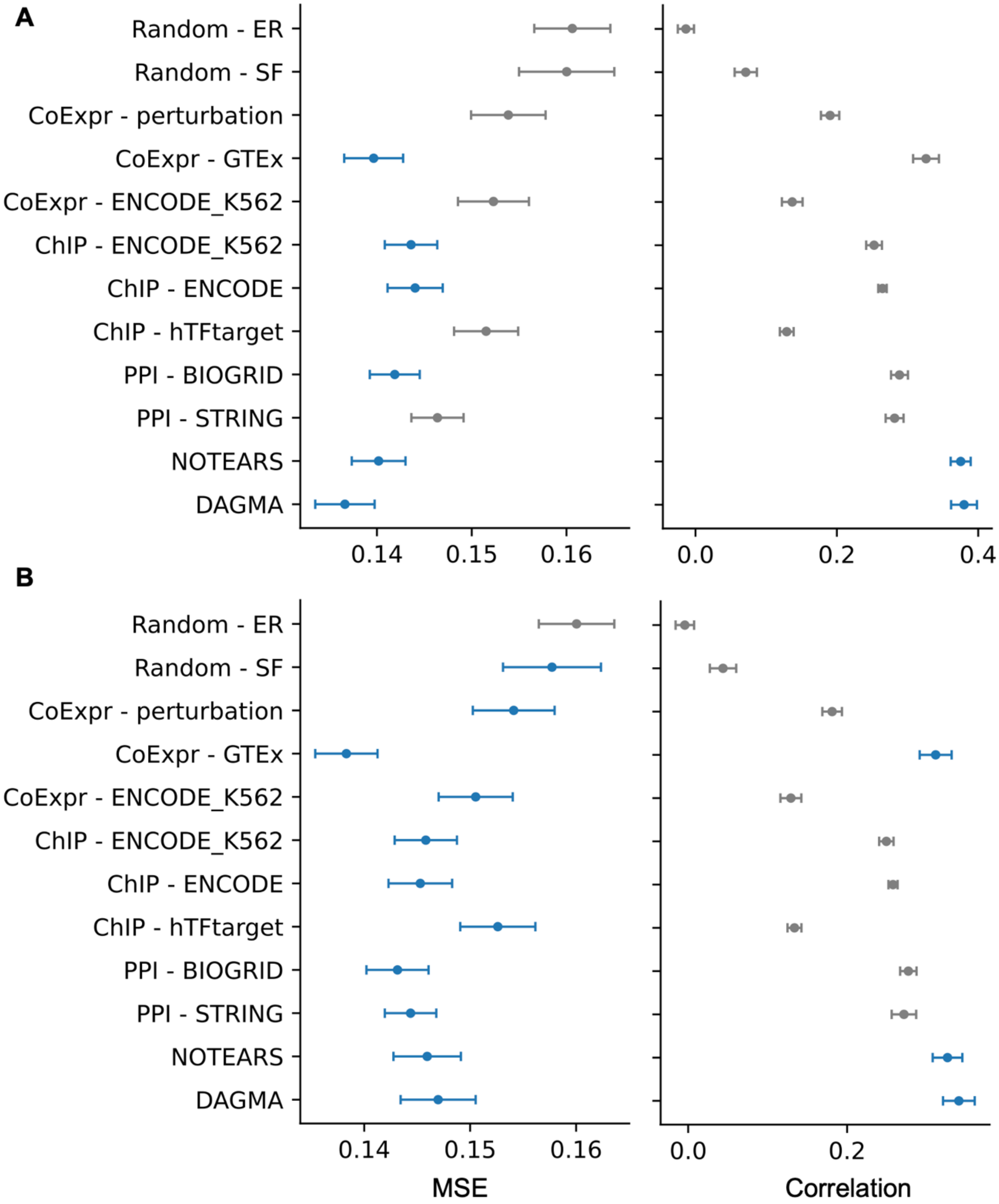

Figure S9. Performance of the representative model when allowing a longer message-passing distance. By adding additional GCN layers (followed by ReLU activation) to the existing GNN structure, the information was passed down further in the networks. The explanation of each network label was the same as those in the caption of Figure 5C. Error bars showed the standard error from 5 different train-test splits and 10 random model initiations for each split, a total of 50 runs. Results were shown in MSE (left) and Pearson correlation (right) for three hops (panel A) and four hops (panel B). Grey color indicated that result was statistically significantly (two-sided Welch's t-test, $p<0.05$) different to the result of DAGMA, while blue color indicated insignificance.

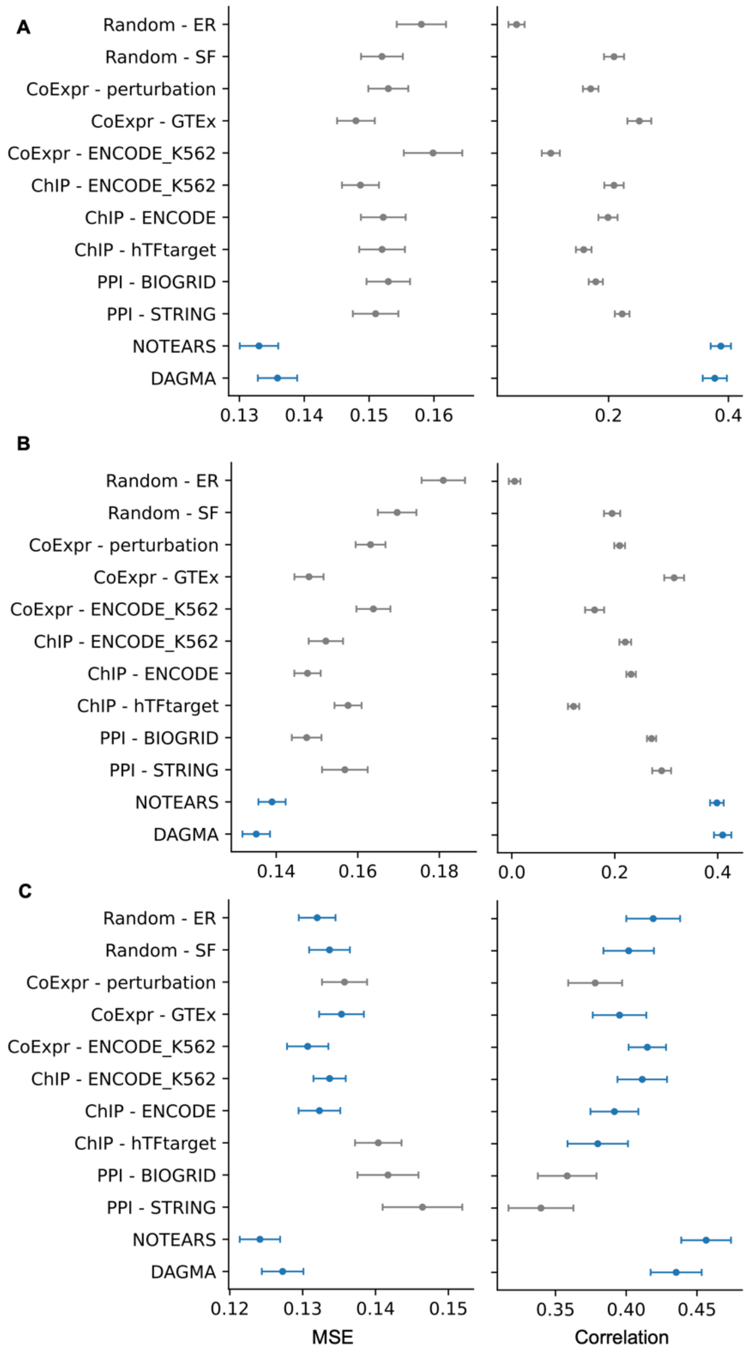

Figure S10. Performance of the representative model with different graph convolution methods. We replaced the GCN layers in the original model (Figure 5B) with graph attention layers (GAT, panel A), combining two GCN layers into one single simplified graph convolution (SGC) layer with message passing depth set to 2 (panel B) or replacing by GraphSAGE layers (panel C). The explanation of each network label was the same as those in the caption of Figure 5C. Error bars showed the standard error from 5 different train-test splits and 10 random model initiations for each split, a total of 50 runs. Results were shown in MSE (left) and Pearson correlation (right). Grey color indicated that result was statistically significantly (two-sided Welch's t-test, $p<0.05$) different to the result of DAGMA, while blue color indicated insignificance.

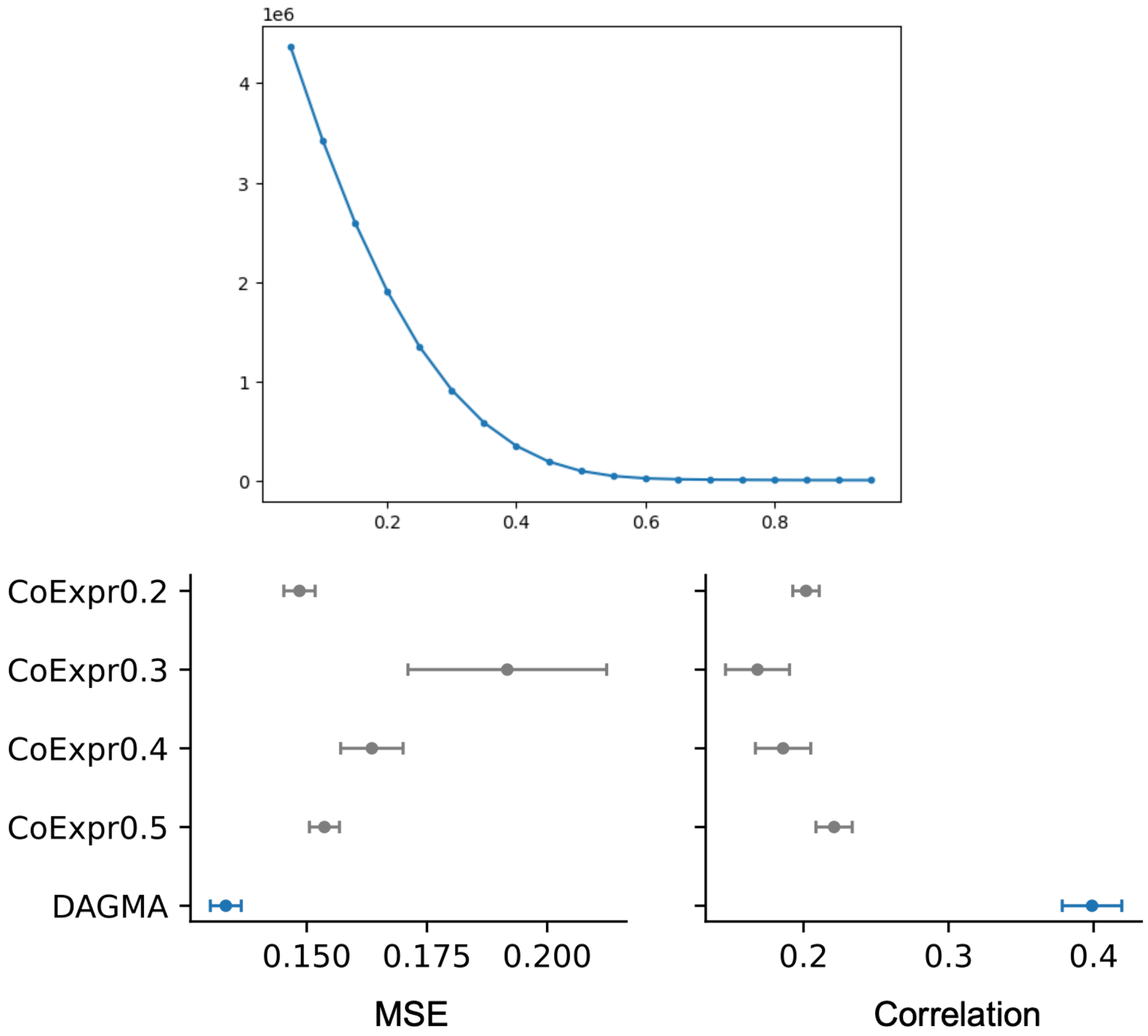

Figure S11. **Changing threshold for co-expression network.** Top: number of edges decreased as the co-expression threshold increase. Bottom: representative task one model using co-expression networks with different threshold.

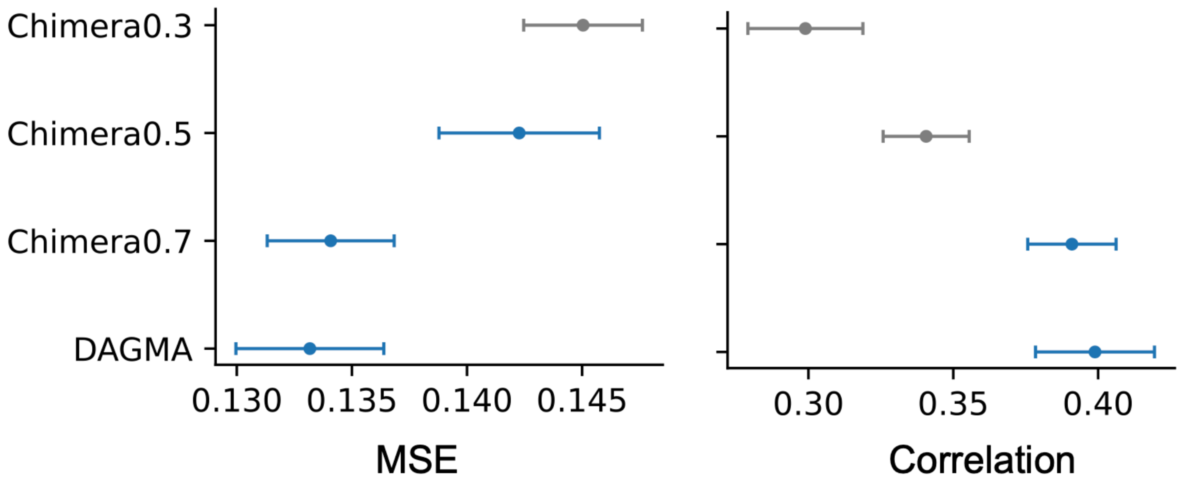

Figure S12. **Performance of the representative model with a chimera network.** We randomly sampled 500 edges from a co-expression network and added those to the DAGMA network to form a chimera network. The value in the name indicated the threshold used when defining the co-expression network. For example, Chimera0.3 indicated that we kept all co-expression value larger than 0.3 when building the co-expression network.
